# Misconceptions About Obesity and Weight Stigma in Brazilian Healthcare Professionals

**DOI:** 10.1111/cob.70074

**Published:** 2026-03-18

**Authors:** Paula Victoria Sozza, Eva Penelo, Stuart William Flint, David Sánchez‐Carracedo, Sebastião Sousa Almeida, Telma Maria Braga Costa, Maria Fernanda Laus

**Affiliations:** ^1^ Departamento de Psicologia Universidade de São Paulo Ribeirão Preto Brasil; ^2^ Departament de Psicologia Clínica i de la Salut Universitat Autònoma de Barcelona Barcelona Spain; ^3^ Curso de Nutrição Universidade de Ribeirão Preto Ribeirão Preto Brazil; ^4^ Departament de Psicobiologia i de Metodologia de les Ciències de la Salut Universitat Autònoma de Barcelona Barcelona Spain; ^5^ School of Psychology University of Leeds Leeds West Yorkshire UK; ^6^ Departamento de Ciências da Saúde Universidade de São Paulo Ribeirão Preto Brazil

**Keywords:** anti‐fat bias, healthcare provider, obesity, weight prejudice

## Abstract

People living with obesity (PLWO) frequently experience weight stigma (WS) in healthcare settings, leading to disparities in treatment. Although research shows that healthcare professionals (HCPs) often hold stigmatising views, this issue remains underexplored in Brazil. The objective of this study is to examine WS and beliefs about obesity amongst Brazilian HCPs. Five hundred seven Brazilian HCPs completed a survey assessing sociodemographic data, beliefs about obesity and the Fat Phobia Scale‐Short Form (FPS‐SF) for assessing WS. Linear regression models analysed the association between these variables. HCPs who are men (*p* = 0.007) and physicians (*p* < 0.001) had significantly higher WS. Participants who attributed overeating to physiological dysfunction (*p* = 0.004) or the food environment (*p* = 0.020) reported significantly lower WS compared to those who attributed it to emotional eating. Those who attributed weight loss difficulty to genetic or metabolic factors (*p* < 0.001) had significantly lower WS compared to those that reported lack of motivation or self‐discipline. People who reported a belief that there are factors beyond patients' control (*p* = 0.001), inadequate care by HCP (*p* = 0.018) or biological mechanisms (*p* = 0.007) that prevent weight loss reported significantly lower WS compared to HCPs who believe it's the result of a lack of motivation/self‐discipline. In conclusion, higher levels of WS were observed in HCPs who are men, physicians and HCPs who attribute the causes and management of obesity to personal responsibility. These findings emphasise the need for interventions to address WS in HCPs and to improve care for PLWO.

## Key Points

- What is already known about this subject.
  ○ Weight stigma (WS) in healthcare settings has been widely documented, with studies indicating that healthcare professionals (HCPs) often hold stigmatising attitudes towards people living with obesity (PLWO).
  ○ These biases can negatively impact quality of care and health outcomes.
  ○ However, most research examining WS has been conducted in North America and Europe, with limited evidence from Brazil.
  ○ Given the growing awareness of WS in healthcare, understanding how these biases manifest in Brazil given the high prevalence of obesity is crucial to addressing disparities in obesity care.
- What this study adds.
  ○ This study is the first to examine WS related to obesity‐related beliefs and sociodemographic information amongst Brazilian HCPs.
  ○ This study provides insights regarding WS in Brazilian HCPs, and how it varies by sociodemographic data such as gender, age and weight status, as well as professional information and beliefs about obesity.
  ○ Our findings reveal that HCPs who are men and physicians exhibit higher levels of WS.
  ○ Additionally, those who provide care for PLWO report lower WS.
  ○ Moreover, attributing obesity to individual blame is associated with higher WS.

## 1 Introduction

Clinical obesity is a chronic disease characterised by excessive or abnormal fat accumulation that poses health risks, and it is associated with multiple causes, including environmental, genetic and health‐related behaviours [1, 2, 3]. Alongside rising obesity rates, weight‐related stigma has also become a pressing concern. In 2020, the Consensus Statement for Ending Stigma of Obesity was published with recommendations to eliminate weight bias. In the Consensus Statement, weight stigma (WS) was defined as the social devaluation and defamation of people living with obesity (PLWO), resulting in negative attitudes, stereotypes, discrimination and prejudice [4]. The framing of obesity as a disease requires careful consideration, as excessive emphasis on individual responsibility can reinforce weight bias and potentially encourage unhealthy weight‐loss behaviours. This perspective may reflect underlying weight bias and stigma [3].

Weight stigma is perpetuated across multiple societal domains, including research, healthcare, media and public policies [5]. PLWO are frequently portrayed using harmful stereotypes, labelled as lazy, undisciplined or lacking intelligence. Such narratives contribute to the misconception that weight is solely a matter of personal control, disregarding the complexity involved [6].

Research on WS indicates that stigmatising attitudes are strongly shaped by beliefs about individual responsibility embedded in broader social ideologies, leading to moralised judgements when culturally devalued attributes, such as fatness, are perceived as controllable. According to attribution‐value models of prejudice, negative affect towards social groups arises when individuals are perceived as responsible for attributes that are culturally devalued. In addition, WS is associated with not only attributions of controllability to individuals with obesity, but also with negative societal perceptions of obesity [7]. Complementarily, the Health Stigma and Discrimination Framework conceptualises stigma as a multilevel process operating across individual, interpersonal, organisational and policy contexts, encompassing its drivers and facilitators, stigma marking, manifestations and outcomes [8]. From a social‐cognitive perspective, internalisation of stigma occurs when individuals become aware of societal stereotypes and apply these beliefs to themselves, a process that, in the context of body weight, results in self‐blame, shame and adverse psychological and behavioural outcomes [9, 10]. Together, these perspectives highlight how repeated exposure to stigmatising narratives can transform external stigma into internalised beliefs that shape both self‐perception and behaviour.

It should therefore come as no surprise that evidence highlights that PLWO often experience teasing, social exclusion and discrimination in various settings. These experiences contribute to poorer socioeconomic conditions, negatively affect physical and mental health [11], and limit access to healthcare, education, career opportunities and social relationships, further marginalising PLWO [6].

Healthcare professionals (HCPs) are important in promoting equitable and patient‐centred care, however, implicit and explicit stigma can undermine healthcare quality. Research has highlighted that HCPs are not immune from WS, associating obesity with patient non‐compliance. Weight stigma may contribute to a lower quality of care provided to PLWO, which may lead to the development of and/or non‐identification and diagnosis of associated disease (e.g., cancers), as well as exacerbation inequalities associated with obesity [11, 12, 13]. Studies reveal that WS significantly influences medical decision‐making, leading to compromised care. Weight stigma in HCPs affects medical consultations, diagnostic procedures and patient interactions, manifesting as blame, judgement, reduced eye contact, less patient‐centred communication, reduced time spent on patient care, decreased confidence in patients' ability to adhere to treatment, incomplete physical examinations and an overemphasis on weight as the primary health concern [4, 12, 13, 14, 15, 16, 17].

These negative attitudes not only diminish trust and adherence to treatment but also contribute to poorer physical and mental health outcomes, including depression, anxiety, stress, disordered eating, substance abuse, low self‐esteem, social isolation and poor quality of life [12, 13]. Weight stigma in healthcare settings acts as a stressor, triggering physiological and behavioural responses such as avoidance of physical activity and unhealthy eating behaviour, further increasing the risk of chronic diseases. Structural barriers, such as inadequately sized medical equipment and limited insurance coverage for obesity treatments, exacerbate these challenges [4, 15, 16, 17].

Beyond the inaccurate beliefs and WS in HCPs reported in previous research, data also highlight emotions expressed by HCPs towards PLWO. Alberga et al. [18] found that family physicians expressed negative feelings about providing care to PLWO. Nearly a quarter felt uncomfortable with the idea of friendship with PLWO, half believed they strain the public healthcare system, and a third reported frustration when treating PLWO; more than a quarter viewed PLWO as non‐compliant with treatment, and the others admitted feeling disgusted by PLWO [18]. In another study, Sobczak and Leoniuk [19] reported that HCPs witness various forms of WS in healthcare settings. Women and younger HCPs were more likely to recognise its prevalence, reporting that weight discrimination is a common issue within the medical system. Many have witnessed inappropriate behaviours, including judgmental comments, disgusted expressions and ironic smirks. They also identified systemic barriers, such as the lack of proper medical equipment, as discrimination. Less experienced professionals are more likely to recognise these issues than those with longer careers [19]. These findings highlight the importance of addressing structural problems, such as discriminatory systems and policies, to ensure fair and equitable treatment for PLWO.

Research indicates that physicians tend to exhibit higher WS than other HCPs. A 2013 study in the United Kingdom (UK) found that medical and nutrition trainees displayed more WS than nursing trainees. Furthermore, individuals with a higher self‐reported body mass index (BMI) and those who recognised obesity as not solely under individual control reported lower WS [20]. Similarly, in a study of Polish HCPs, Baska et al. [21] found that physicians demonstrated higher levels of WS and reported greater discomfort and impatience when interacting with PLWO, whereas dietitians showed greater empathy [21].

A multinational study published in 2020 evaluated WS and biassed beliefs about obesity amongst HCPs from 77 countries across the world and the general population from the UK, Australia, New Zealand and the United States of America (USA). Findings indicated that whilst HCPs exhibited slightly lower WS than the general population, both groups held stigmatised beliefs associating obesity with health‐related behaviours and personal responsibility. Furthermore, participants who attributed individual causes to obesity had higher WS [22]. This finding is consistently reported, where the widespread belief is that obesity is the result of a lack of willpower and an issue of personal responsibility, and where all individuals can either prevent or manage their weight solely through health‐related behaviours [11, 22].

A recent review of WS in Latin America, Asia, the Middle East and Africa highlighted the scarcity of data from these regions [23]. In Latin America, only 45 studies were identified, primarily from Brazil, and conducted between 2013 and 2020. They addressed several important topics related to WS; however, there were few that examined WS in healthcare and social contexts [23]. Of the few that have been conducted, research in Brazil has explored the experiences of dietitians living with obesity in the workplace, revealing that participants faced stigma and distress in both professional settings and broader social interactions [24], as well as public perceptions and stereotypes about their competence, indicating that dietitians with obesity were perceived as warm but less competent [25]. Other research investigated women's experiences with body image and discrimination in a non‐prescriptive, multidisciplinary intervention [26], as well as efforts to adapt scales related to WS [27].

There are a growing number of educational initiatives aimed at reducing WS in the international literature; however, outcomes remain inconsistent, with interventions often characterised by methodological limitations and formats that insufficiently capture the complexity of real‐world healthcare settings. Accordingly, there has been increasing advocacy for educational strategies that operate across multiple levels and incorporate the social, cultural and subjective dimensions of obesity [28, 29]. Within this context, educational strategies have increasingly drawn on psychological frameworks to address WS, including attribution‐value theory and beliefs about controllability, which help explain how emphasising personal responsibility reinforces stigmatising attitudes [30, 31]. Interventions have also incorporated empathy‐evoking and dramatisation‐based approaches, such as patient narratives and experiential exercises, to challenge implicit biases and foster perspective‐taking. In addition, weight‐inclusive approaches that shift the focus from weight control to health, well‐being, and respectful care have been proposed, often combined with mixed educational techniques integrating didactic content, reflective practise and experiential learning. Together, these strategies aim to move beyond purely informational models and address the underlying beliefs and emotional processes that sustain WS amongst HCPs [30].

In Brazil, formative experiences grounded in this perspective have shown promise in fostering broader understandings of obesity, encouraging critical reflection on professional practises and incorporating the voices and lived experiences of PLWO [29, 32]. However, evidence regarding the outcomes and impacts of such interventions in the Brazilian context remains limited, underscoring the need to sustain and expand these efforts and to systematically evaluate their medium‐ and long‐term repercussions. The paucity of research examining WS in Latin and South America was also highlighted in a 2024 call [33], where WS in healthcare and its impact on practise was highlighted as a cause for concern that required investigation.

There is a significant lack of data on WS in Brazil amongst HCPs and health contexts. This gap underscores the importance of investigating this issue in Brazil, contributing to the broader understanding of WS in Latin American countries and other low‐ and middle‐income regions, which remain underrepresented in the literature compared to studies from the USA and Europe. In line with the World Obesity Federation's recommendation to expand WS research globally, particularly in different economical contexts and languages, this study intends to address part of this critical gap [34].

Thus, this study aimed to examine (1) obesity‐related beliefs and WS, specifically evaluating fat phobia in Brazilian HCPs, and (2) WS by profession, sociodemographic data and obesity beliefs.

Based on previous studies of WS in HCPs, this study hypothesised that HCPs who are men, younger HCPs, physicians, and those who do not directly provide care for PLWO are more likely to report higher WS than women, older HCPs, other professions and HCPs who directly care for PLWO respectively. Additionally, it is expected that HCPs who attribute obesity primarily to individual responsibility will report higher WS compared to those who do not attribute obesity primarily to individual responsibility.

## 2 Methods

This study used a cross‐sectional, online survey design to recruit Brazilian HCPs. Invitations were sent to potential participants to take part in the study between March 2022 and January 2024. Participants were recruited via email, and advertisements were posted on social media. Various health and educational institutions disseminated the invitations to their HCPs, whilst the researchers also shared and promoted the survey on social media. Potential participants received information about the study, and those who agreed to participate provided digital informed consent before completing the survey online, which was hosted on the REDCap platform.

The sample size was defined by convenience. A snowball sampling method was employed, and participants were encouraged to refer the study to others. Inclusion criteria were being an adult and self‐identifying as a Brazilian healthcare professional. Participants who were under 18 years old or who reported professions not related to healthcare were excluded. Initially, 739 persons identified as ‘healthcare professionals’ agreed to participate in the study. However, data from 18 participants were excluded as they listed their professions as veterinary medicine (*n* = 8), animal science (*n* = 1), telecommunications (*n* = 1), tantric therapy (*n* = 1) and biology (*n* = 5). Additionally, data from 113 participants were excluded for leaving all the survey data blank; 97 answered only the demographic questions without completing the other instruments, five provided only region and age, and one responded only the region. Two participants did not report their age but were retained in the sample as they were considered adults based on their sociodemographic data. Thus, the final sample comprised 507 HCPs (Table 1).

**TABLE 1:** Demographic characteristics of the total sample.

Characteristics	*n*	M	SD	Minimum	Maximum
Age (years)	505	37.6	12.3	18.0	73.0
BMI (kg/m^2^)	495	27.7	6.5	14.9	54.6
FPS‐SF total score	478	3.3	0.7	1.0	5.0

				*n*	%
Age	< 25 years			68	13.5
	25–34 years			172	34.1
	35–44 years			134	26.5
	45–54 years			69	13.6
	≥ 55 years			62	12.3
Gender	Women			394	78.5
	Men			105	20.9
	I prefer not to answer			3	0.6
Sexual orientation	Heterosexual			406	81.2
	Homosexual			32	6.4
	Bisexual			49	9.8
	Pansexual			9	1.8
	Other			4	0.8
Skin colour^a^	White			397	79.2
	Brown			27	5.4
	Black			70	14.0
	Yellow			6	1.2
	Indigenous			1	0.2
Marital status	Single			229	45.8
	Married			177	35.4
	Stable relationship			60	12.0
	Divorced			28	5.6
	Separated			5	1.0
	Widower			1	0.2
Highest educational degree	Graduation			146	29.4
	Technician			26	5.2
	Master's			100	20.1
	Doctorate/PhD			51	10.3
	Post‐doctorate			10	2.0
	Specialisation/improvement/residency			164	33.0
Profession	Physician			84	16.8
	Dietitian			131	26.1
	Psychologist			61	12.2
	Nursing professional			61	12.2
	Complementary HCPs			68	13.6
	Other			96	19.1
Provide care for PLWO	Yes			298	59.6
Monthly income^b^	Less than one minimum wage			26	5.2
	One to three minimum wages			199	39.7
	Four to five minimum wages			81	16.1
	More than five minimum wages			196	39.0
Regions of Brazil	Northern			11	2.2
	Northeastern			36	7.1
	Central‐Western			19	3.8
	Southeastern			379	74.8
	Southern			61	12.1
Weight status	Underweight or normal weight (< 25 kg/m^2^)			207	41.8
	Overweight (> 25 < 30 kg/m^2^)			142	28.7
	Obesity (≥ 30 kg/m^2^)			146	29.5
Recent weight changes	Gained weight			174	35.0
	Lost weight			122	24.6
	No changes			201	40.4
Obesity treatment	No			386	78.3
Weight concerns	Not concerned about weight and have no plans to lose weight in the next 6 months			143	29.0
	Concerned about weight but have no plans to lose weight in the next 6 months			30	6.1
	Aware of being overweight and seriously considering taking steps to lose weight			92	18.7
	Aware of being overweight and intending to take steps to lose weight in the next month			53	10.7
	Involved in or enrolled in a weight loss plan			95	19.3
	Have managed to lose weight in the last year and have kept it off			52	10.5
	Have managed to lose weight in the last year but have not been able to keep it off			28	5.7

Abbreviations: BMI = body mass index; FPS‐SF = fat phobia scale‐short form; HCPs = healthcare professionals; PLWO = people living with obesity.

^a^ Race/ethnicity categories by the official Brazilian census.

^b^ The minimum wage was R$1212.

A pilot test was conducted at the beginning of the study with volunteer HCPs to assess the data collection process and time, which took approximately 30 min on average. The response duration after the start of data collection was not evaluated in this study. No incentives were offered for participation, which was voluntary.

### 2.1 Outcomes and Variables

Participants' characteristics were collected through questions about age, gender, sexual orientation, skin colour, marital status, highest educational degree, profession, whether they provide care for PLWO or not, monthly income and self‐reported height and weight. BMI was calculated from self‐reported height and weight to classify weight status. Additionally, three questions assessed weight changes and weight loss in the past 3 months, and concerns about body weight (‘Have you experienced any weight change in the past 3 months?’; ‘In the past year, have you received or are you currently receiving professional treatment for overweight or obesity?’ and ‘Which of the following statements best describes you today?’).

The professions were originally presented in 19 categories: general practitioners and family physicians (primary care), obesity specialists, metabolic bariatric surgeons, endocrinologists, internal medicine physicians, psychologists, psychiatrists, dietitians, other HCPs, commissioners, public health specialists, healthcare managers/administrators, occupational therapists, speech therapists, physiotherapists, physical education professionals, nursing technicians, nursing assistants and a final category labelled ‘other’ to write a different option. Due to the low number of respondents in some categories, the professions were later grouped to allow for more effective statistical analysis. The grouped categories included: physicians, psychologists, dietitians, nursing professionals, complementary HCPs who work directly with patients (such as physiotherapists, occupational therapists, pharmacists, speech therapists and physical education professionals), and the ‘other’ category combining respondents who selected ‘other’ as well as administrative professionals.

To assess biassed beliefs about obesity, the Attitudes, Stigma and Knowledge (ASK) on Obesity survey was used that consists of questions developed by O'Keeffe and colleagues [22] through a collaborative consensus, exploring perceptions of obesity, attitudes towards obesity and knowledge of related therapies for obesity. These questions were translated to Brazilian Portuguese, and each item was described based on response frequencies. We focused the main analysis only on the seven questions addressing personal responsibility for obesity, as these are interpersonal WS representations (questions 2, 3, 4, 6, 7, 12 and 13; e.g., ‘What makes it difficult for people to lose weight?’). The other questions are about diabetes, metabolic surgery, referring patients for metabolic bariatric surgery, funding for obesity research and perceptions of weight stigma.

Weight stigma was measured using the Fat Phobia Scale (FPS)—Short Form (FPS‐SF) [35] adapted for the Brazilian population [27]. The FPS‐SF presents 14 pairs of opposite adjectives describing PLWO (e.g., ‘lazy‐industrious’, ‘no will power—has will power’, ‘*attractive ‐unattractive*’). Responses are recorded on a five‐point Likert type scale (1–5). After reversed when necessary (items; 1, 2, 8, 9, 11, 13 and 14), the total score is obtained by adding the scores for all items and dividing this by 14 (the number of items). The cultural adaptation of the scale followed established guidelines and included forward translation by two independent translators, synthesis, expert committee review composed of three specialists in the field of obesity and/or WS, pilot study with 20 people from the target population and back‐translation. Equivalence across semantic, idiomatic, cultural and conceptual dimensions was evaluated, and the back‐translated version was approved by the original authors. Full methodological details of the adaptation and validation process are available in the original publication describing these procedures [27].

However, although the FPS‐SF was originally adapted for use in Brazil with 14 items, factor analyses conducted with our sample indicated that a one‐factor and 11‐item model showed a more favourable internal structure. Item 10 showed low factor loadings in both exploratory factor analysis (EFA) and confirmatory factor analysis (CFA) (< 0.30), whilst items 13 and 14, despite moderate loadings in the first factor, also loaded onto a second factor, evidencing that both behaved differently from the rest of the items (Table S1). Internal consistency coefficients were high for both potential versions (*α* = 0.87 with 14 items; *α* = 0.86 with 11 items), supporting the use of the shorter version, where all items contributed to the internal consistency reliability of the total score. Therefore, we opted to use the 11‐item version in our analyses.

### 2.2 Statistical Analysis

Analyses were performed with the SPSS23.0 and Stata18.0 programmes. Descriptive analyses and frequency distributions were conducted to characterise the sample and the frequency responses about obesity beliefs. Multiple linear regression analysis was performed to examine the association between WS based on FPS‐SF scores as criterion and several predictors, including sociodemographic variables, factors related to weight status and body weight and the ASK questions about obesity. Each of the models was adjusted by the potential confounding variables (demographic characteristics). For the regression models related to the seven ASK questions and the three questions related to weight changes, weight loss in the past 3 months, and concerns about body weight, covariates were included when the difference between parameter estimates of adjusted model and the crude model was greater than 10% [36]. Reference categories for categorical predictors were selected based on those identified in the literature as the most weight‐stigmatising. The significance level was set at *p* < 0.05.

### 2.3 Ethics Statement

Approval for the study was obtained from the University Institutional Review Board (IRB; no. 51927421.1.0000.5498). All participants provided online informed consent before data collection. Respondents were informed about the voluntary nature of their participation and the measures taken to ensure the confidentiality and anonymity of their data. They were also notified of their right to decline to answer any questions or withdraw from the survey at any point.

## 3 Results

**FIGURE 1:**
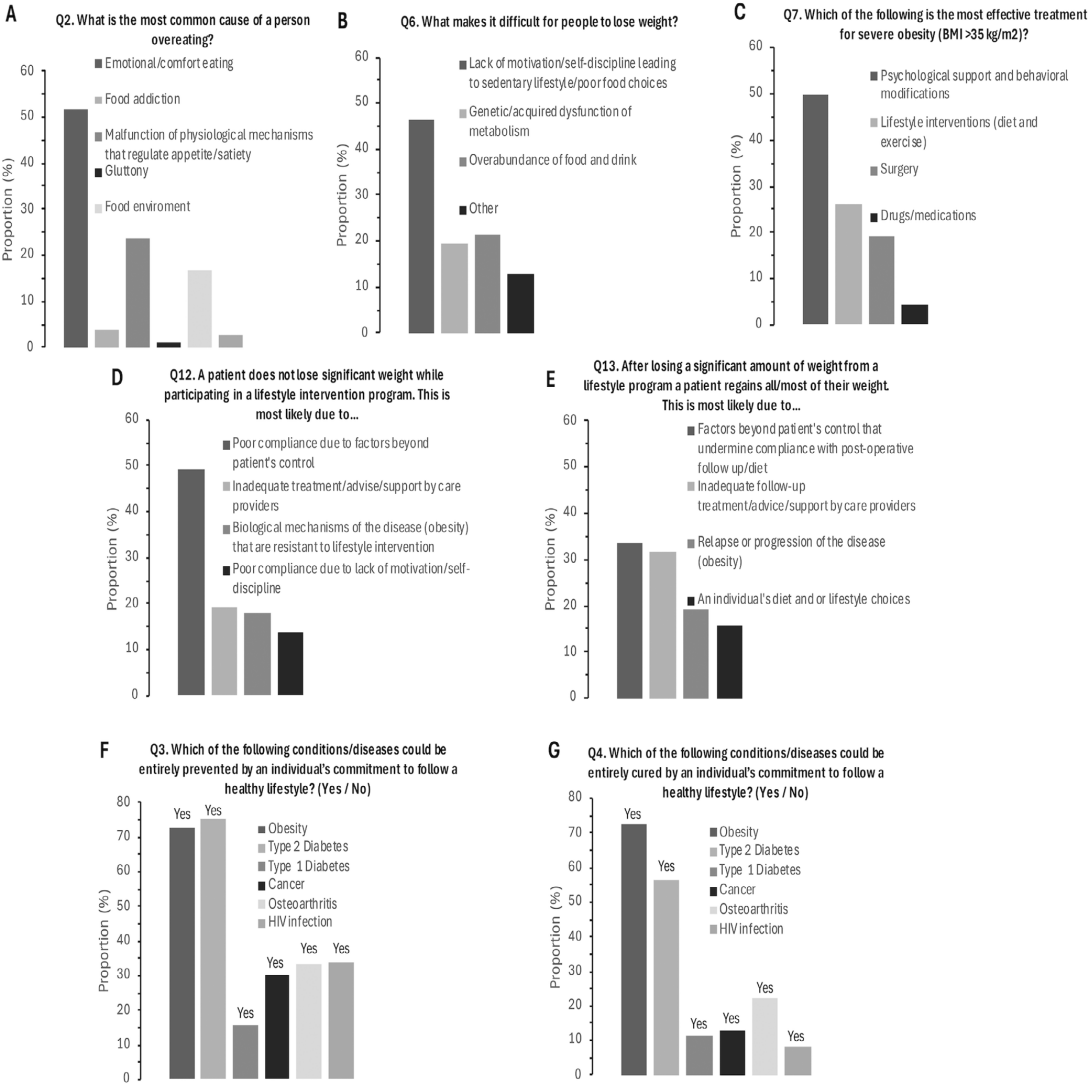
Description of the beliefs about obesity from the ASK questionnaire in Brazilian HCPs. Graphs (A–E) each refer to a single question with four to six response options. In contrast, graphs (F) and (G) represent subcategories of the questions, where participants selected ‘yes’.

HCPs predominantly endorsed individual and behavioural explanations for obesity, such as emotional eating, lack of motivation, and unhealthy lifestyle behaviours. Psychological and behavioural interventions were viewed as the most effective treatments for severe obesity. Although some recognised systemic barriers to weight management, many still believed that obesity is fully preventable and curable through health‐related behaviours changes (Figure 1).

The means and standard deviations of the FPS‐SF according to sociodemographic variables and the seven ASK questions are presented in the Supporting Information (see Table S2). In regression analysis, significantly higher WS scores were associated with being a man, not providing care to PLWO, and being a physician compared to dietitians, psychologists and other HCPs professions. No significant differences were found between physicians and nursing professionals or complementary HCPs, nor were there age‐related differences (Table 2, first part). Additionally, HCPs who were aware of being overweight and planning to lose weight in the next month or who were currently enrolled in a weight loss plan had significantly higher WS scores compared to those who were not concerned about their weight (Table S3).

**TABLE 2:** Linear regression results of factors related to fat phobia scores.

Outcome	Effects	*B* (95% CI)	*p* ^a^
Stigma	Gender: Women	−0.28 (−0.45; −0.11)	0.001
	Provide care for PLWO: Yes	−0.19 (−0.32; −0.05)	0.006
	Profession (reference category: Physician/Doctor)		*< 0.001*
	Dietitian	−0.53 (−0.75; −0.32)	< 0.001
	Psychologist	−0.29 (−0.54; −0.04)	0.022
	Nursing professional	−0.23 (−0.49; 0.02)	0.073
	Complementary HCPs	−0.01 (−0.25; 0.23)	0.927
	Other	−0.35 (−0.57; −0.13)	0.002
	Weight status (reference category: BMI < 25 kg/m^2^)		*0.452*
	Overweight (BMI between 25 and < 30 kg/m^2^)	−0.07 (−0.23; 0.08)	0.355
	Obesity (BMI > 30 kg/m^2^)	0.03 (−0.013; 0.019)	0.737
	Age group (reference category: aged < 25 years)		*0.674*
	25–34 years	0.13 (−0.07; 0.33)	0.213
	35–44 years	0.10 (−0.11; 0.31)	0.351
	45–54 years	0.18 (−0.07; 0.43)	0.159
	> 55 years	0.14 (−0.12; 0.40)	0.303
Stigma^b^	Q2: Cause of overeating (reference category: Emotional/comfort eating)		*0.019*
	Food environment	−0.21 (−0.40; −0.02)	0.030
	Food addiction	0.03 (−0.33; 0.38)	0.878
	Malfunction physiological mechanisms	−0.25 (−0.42; −0.09)	0.002
	Other	−0.04 (−0.48; 0.40)	0.858
Stigma^c^	Q3: Individual commitment prevents obesity: No	−0.25 (−0.40; −0.10)	0.001
Stigma^c^	Q4: Individual commitment cures obesity: No	−0.31 (−0.46; −0.16)	< 0.001
Stigma^c^	Q6: Difficulty to lose weight (reference category: Individual lack of motivation/self‐discipline sedentary lifestyle)		*< 0.001*
	Not modifiable (genetic and/or metabolism)	−0.38 (−0.56; −0.21)	< 0.001
	Overabundance of products promoting weight gain	−0.05 (−0.23; 0.12)	0.541
	Other	−0.20 (−0.41; 0.01)	0.059
Stigma^d^	Q7: Treatment most effective for severe obesity (reference category: Lifestyle interventions [diet, exercise])		*0.021*
	Medications	0.10 (−0.23; 0.42)	0.554
	Psychological support and behavioural modifications	−0.09 (−0.25; 0.07)	0.265
	Surgery	0.19 (−0.01; 0.38)	0.064
	Q7 (reference category: Surgery)^e^		*0.021*
	Psychological support and behavioural modifications	−0.28 (−0.45; −0.10)	0.002
Stigma^f^	Q12: Cause for not losing significant weight whilst participating in a lifestyle intervention programme (reference category: Poor compliance due to lack of motivation/self‐discipline)		*0.002*
	Poor compliance beyond patient's control	−0.40 (−0.62; −0.19)	< 0.001
	Inadequate aspects by care providers	−0.32 (−0.57; −0.07)	0.011
	Biological mechanisms of obesity	−0.39 (−0.64; −0.15)	0.002
Stigma^c^	Q13: Cause for regaining weight after losing from a lifestyle programme (reference category: Individual's diet and lifestyle choices)		*< 0.001*
	Factors beyond patient's control	−0.50 (−0.70; −0.30)	< 0.001
	Inadequate aspects by care providers	−0.41 (−0.61; −0.20)	< 0.001
	Relapse/progression of the disease	−0.36 (−0.59; −0.14)	0.001

*Note:* The detailed means and SDs are provided in Table S3.

Abbreviations: BMI = Body Mass Index; CI = confidence interval; HCPs = Healthcare professionals; PLWO = people living with obesity.

^a^ In italics: test for the overall effect of categorical predictors with more than two categories.

^b^ Adjusted for all demographics (gender, assists, profession, weight status and age group); category ‘gluttony’ excluded because of extremely low endorsement (*n* = 5).

^c^ Adjusted for profession.

^d^ Adjusted for gender, care provision for PLWO and profession.

^e^ Given that the overall effect was statistically significant, an additional pair‐wise comparison was conducted, with surgery as the reference category.

^f^ Adjusted for gender, care provision for PLWO, profession and weight status.

Regarding ASK questions, significantly lower WS scores were reported by those attributing overeating to the food environment or physiological malfunction, compared to emotional/comfort eating. In contrast, significantly higher WS scores were associated with believing that a healthy behaviour can prevent or cure obesity. Attributing difficulty in losing weight to modifiable factors (e.g., lack of motivation/self‐discipline) was also significantly associated with higher WS scores than citing genetics and metabolism. Those who considered surgery as the most effective treatment for severe obesity exhibited significantly higher WS scores than those who reported psychological or behavioural approaches. Similarly, attributing failure to lose weight or weight regain to individual lack of motivation/self‐discipline was significantly associated with higher WS scores than attributing it to factors beyond the patient's control in both cases, to biological mechanisms for failure to lose weight, to disease relapse for weight regain or to inadequate care by an HCP in both cases (Table 2, second to eighth part). Similar patterns were observed using the full 14‐item FPS scores (Table S4).

## 4 Discussion

The findings of this study provide important insights into obesity‐related beliefs and WS amongst Brazilian HCPs. Overall, HCPs mainly attributed both the causes and the management of obesity to personal responsibility. Nevertheless, many believed that obesity is fully preventable and curable through changes in health‐related behaviours, despite acknowledging some systemic barriers. Moreover, the results highlight high levels of WS, particularly amongst certain sociodemographic and professional groups, including men, physicians and HCPs who do not provide care to PLWO. Higher WS was also identified amongst HCPs who perceived themselves as having overweight and amongst those planning or actively engaged in weight‐loss efforts. In addition, biassed beliefs about the causes, prevention and treatment of obesity were associated with higher WS levels.

Findings using the FPS‐SF to assess WS have been observed in other countries, including the multinational study of 1567 HCPs from 77 countries on which this study is based [22]. That study reported a FPS‐SF of 3.4; a German study with 49 dietitians reported 3.35 [37]; an Australian study with 201 dietitians, 3.37 [38]; a study with general practitioners, 3.7 [39]; and one with 285 certified physical therapists and 115 students, 3.6 [40]. These scores, along with ours, reflect high levels of fat phobia amongst HCPs, raising concerns about healthcare quality for PLWO.

Although most participants were predominantly younger and were classified as living with overweight or obesity, WS levels did not significantly differ across age or weight status categories. However, confirming the hypothesis, men showed higher WS than women, and those not providing care for PLWO were more fatphobic. These findings are consistent with studies showing that women appear more attentive to weight stigma [19], express less WS than men [18, 22, 39], and perceive more discrimination against PLWO and report more positive interactions with them [21]. Moreover, analysis revealed variations amongst different medical professions, with physicians exhibiting the highest WS compared to dietitians, psychologists and others. Moreover, providing care to PLWO is associated with lower WS. And some studies also suggest higher BMI is linked to lower WS, whilst those with lower BMI agree more with stigmatising attitudes [18].

Physicians tend to show more discomfort and negative attitudes towards PLWO [20, 21]. This mirrors findings from the USA, where medical doctors scored high on implicit and explicit WS, particularly men. WS levels also varied by weight status, with higher WS amongst individuals without obesity [41]. Similarly, UK trainees with higher BMI had modestly lower WS [20].

We also found that HCPs concerned about their own weight and either planning to lose weight or currently in a weight loss programme reported higher WS. From a psychological perspective, this association may be understood in light of processes such as self‐stigma [42, 43] and body moralization [30]. Although internalised WS and thinness ideal were not directly assessed in this study, these processes may help explain the observed interpersonal manifestations of stigma, whereby societal ideals of thinness and personal responsibility are internalised and subsequently reinforced [7]. Professionals who are themselves striving to lose weight may be more likely to endorse moralised beliefs about weight loss, effort and self‐control, which can be projected onto others in clinical contexts [30]. This interpretation highlights the clinical relevance of the finding, as it suggests that personal weight‐related experiences and internalised beliefs amongst healthcare professionals may shape their attitudes towards patients, reinforcing stigmatising care practises.

From a theoretical perspective, our findings as a whole are consistent with the attribution‐value model [7] and stigma internalisation model [9, 10], which highlight how beliefs centred on individual responsibility and controllability contribute to the emergence and maintenance of WS. When body weight is framed as a personally controllable and morally valued attribute, stigmatising attitudes are more likely to be endorsed and, in some cases, internalised. Complementarily, multilevel frameworks conceptualise WS as a process shaped by broader social and institutional contexts [8], reinforcing its implications for healthcare practise.

These findings are important, as characterising the profile of HCPs who are more likely to display WS towards PLWO is an urgent need, allowing for more targeted approaches and intervention strategies. It is already known that women tend to be more attentive to such issues; therefore, it is worth directing training initiatives from undergraduate education onward, as well as continuing education strategies that emphasise men professionals and medical fields, especially in physicians. Additionally, fostering closer engagement of HCPs in the care of PLWO may help reduce WS, since greater contact is associated with lower WS levels.

This study also found that Brazilian HCPs in our sample endorsed several biassed beliefs about obesity showing that they reinforce an emphasis on individual responsibility. WS was significantly higher amongst those who believed overeating stems from emotional eating, food addiction or other personal causes, compared to those citing food environment or physiological malfunction. Similar findings in a prior study [22] suggest that emphasising emotional or comfort eating may contribute to increased individual blame and reinforce negative stereotypes surrounding obesity. It is important to highlight obesity's multifactorial nature, including genetic, environmental, psychological, nutritional and metabolic factors, that affect bodily responses and the regulation, distribution and function of adipose tissue. In particular, the role of the food environment, like the easy access to highly energy‐dense products, is a key factor associated with obesity [3, 6].

Many HCPs believed obesity is entirely preventable and curable through health‐related behaviours changes, a pattern also seen with Type 2 diabetes. Regressions models showed that HCPs holding this belief exhibited higher WS scores. Both conditions are often stigmatised, with individuals blamed for their health issues [44]. Whilst percentages differ, both this and a previously study [22] show that most HCPs emphasise individual health‐related behaviours as causal.

Most HCPs considered changes in health‐related behaviours, such as psychological and behavioural interventions, along with diet and exercise, as the best treatment for severe obesity. Although these approaches support general health, seeing them as sufficient reinforces the idea that obesity is a matter of personal effort [3, 6]. Other countries reported lower endorsement of health‐related behavioral interventions as the main treatment and showed surgery as the best option [22].

Despite the availability of effective options like medications and metabolic bariatric surgery, severe clinical obesity remains undertreated. Metabolic bariatric surgery is recommended for patients with BMI ≥ 35 kg/m [2], regardless of comorbidities, yet many patients only receive advice on health‐related behaviours [45].

Interestingly, in this study HCPs who viewed surgery as the best treatment exhibited higher WS than those favouring behavioural/psychological approaches. This may reflect Brazil's ongoing debates, where surgery is often viewed as an aesthetic procedure rather than a medical necessity, shaped by media portrayals and the weight‐normative discourse [46, 47]. This framing reinforces the idea of fatness as a disease and surgery as an individual‐centred intervention focused on weight loss and a form of “body correction” rather than being a medical recommendation for individuals with severe obesity, explaining its association with higher WS.

This reflects a broader paradox: despite severe obesity impairments, patients often lack access to adequate care unless comorbidities are present, reinforcing systemic barriers to obesity care [3]. Holmes et al. [48] reported low referral rates for surgery, minimal provider discussion, limited dietitian referrals and few pharmacological prescriptions. Controversially, specialist physicians were less likely than primary care providers to guide proper treatment. Barriers such as time constraints, referral difficulties, lack of confidence in making recommendations, alongside WS, likely contribute to inadequate care [48].

Most participants believed that difficulties in losing or maintaining weight result from external factors or inadequate care by HCPs. However, HCPs with higher WS attributed these issues to a lack of motivation or self‐discipline. This oversimplified ‘eat less, move more’ mindset disregards obesity's complexity, weakening public health responses. Recognising obesity as a multifaceted disease is crucial to developing stigma‐free care and policies [6].

This study has limitations. First, it is not representative because participants were mainly young, white, heterosexual women, with high income levels, from southeastern Brazil and these states are amongst the most populous in Brazil, accounting for a significant portion of the country's total population [49] and although efforts were made to recruit HCPs from diverse professional and demographic backgrounds, the sample is not representative of the wider HCP population. Second, data collection was prolonged to reach geographically diverse professionals, but the high number of dietitians may relate to the researchers' networks. Third, as a cross‐sectional study, causality cannot be determined. Fourth, social desirability bias may have led to underreporting of stigmatising beliefs.

Nevertheless, the study has several strengths, offering novel insights from a broad sample of Brazilian HCPs. It is the first to examine beliefs and WS attitudes amongst this population, which is a group underrepresented in literature, and provides valuable evidence given the national obesity burden and anecdotal reports of WS in healthcare.

Finally, this study provides empirical evidence that Brazilian HCPs are not immune to WS attitudes. The majority of Brazilian HCPs in this study reported a belief that obesity can be entirely prevented and treated through a commitment to a healthy behaviour, emphasising a focus on individual responsibility. This is not aligned to current evidence that demonstrates the multifactorial complexity of obesity, where factors outside, and partially outside of a person's control, are evidenced to also impact weight status and contribute to obesity. This emphasis, combined with a biassed understanding of its causes and effective interventions, may contribute to the marginalisation of PLWO and may compromise the quality of care provided. Educational initiatives and healthcare policies must address WS, fostering a broader, evidence‐based understanding of this condition. Strategies to mitigate the impact of these beliefs may include training programmes for HCPs, science‐based clinical guidelines, and patient‐centred approaches, ensuring fair, effective and non‐discriminatory care. In this regard, interventions grounded in psychological theory may be particularly valuable, as they target attributional beliefs, perceived controllability and emotionally rooted assumptions that underlie stigmatising attitudes. Educational efforts that combine cognitive components with empathy‐based, experiential, weight‐inclusive approaches, fostering self‐awareness of WS and promoting a bias‐free culture, appear better suited to address the complexity of WS in healthcare settings, especially when implemented through multifaceted and sustained training initiatives [28, 30, 31].

Furthermore, the findings emphasise the need for continuous monitoring of fat phobia and WS levels amongst HCPs. This ongoing assessment would enable the development of more effective strategies to mitigate these biases in clinical practise in Brazilian contexts. There is an urgent need for targeted interventions, including educational programmes and institutional policies, to dismantle WS in healthcare settings and promote equitable, patient‐centred healthcare of equal quality for all individuals, as already proposed in Brazilian studies [29, 32], future research should explore long‐term interventions and assess their impact on reducing weight bias and improving patient care. Based on this information, several key recommendations can be proposed to address WS in HCPs practises (Box 1).

BOX 1 **Key recommendations to address WS amongst HCPs.**

- Integrate WS education into undergraduate and continuing training programmes, emphasising the deconstruction of individual blame and the multifactorial nature of obesity.
- Ground educational and training initiatives in psychological frameworks (e.g., attribution theory, controllability beliefs, empathy‐based and experiential approaches and weight‐inclusive care) to address the underlying beliefs and emotional processes that sustain WS amongst HCPs.
- Provide targeted training for male professionals, physicians and those in medical fields with limited exposure to patients with obesity.
- Promote direct and meaningful contact between undergraduate students, HCPs and PLWO, as such engagement is linked to lower stigma levels.
- Perpetuate a person‐centred approach to care that moves beyond simplistic ‘eat less, move more’ messages, focusing instead on comprehensive, compassionate and individualised healthcare.
- Broaden understanding of obesity's determinants, including biological, psychological, social and environmental factors.
- Support continuous monitoring and research on WS amongst HCPs to guide effective educational and policy actions.
- Implement institutional policies that ensure discrimination‐free environments and promote respectful, empathetic and equitable care.
- Encourage public policies, universities and healthcare institutions to address individual blame and promote a broader and systemic understanding of obesity.

## Author Contributions

P.V.S. was involved in the study's conceptualization, data curation, investigation, methodology, project administration, drafting the original manuscript and reviewing and editing the final version. E.P. contributed to the formal analysis and methodology, original manuscript drafting and final version review and editing. S.W.F. provided supervision and final manuscript's conceptualization, reviewing and editing. D.S.C. contributed to the final manuscript's conceptualization, methodology, supervision, funding acquisition and reviewing and editing. S.S.A. was responsible for funding acquisition and participated in reviewing the final version. T.M.B.C. contributed to the final manuscript's conceptualization, methodology, supervision, funding acquisition and reviewing and editing. And M.F.L. contributed to the final manuscript's conceptualization, methodology, supervision, funding acquisition and reviewing and editing.

## Funding

This study was supported by the Coordenação de Aperfeiçoamento de Pessoal de Nível Superior (CAPES) (Process No. 88887.514232/2020‐00; 88887.885714/2023‐00; 88887.994538/2024‐00), which provided a doctoral scholarship; and the Conselho Nacional de Desenvolvimento Científico e Tecnológico (CNPq; Process No. 404975/2023‐2), which funded equipment and scientific events. None of these funding sources had any involvement in the design, data analysis, interpretation or writing of this study.

## Conflicts of Interest

P.V.S. declares researcher‐led grants for doctoral scholarship received from the CAPES‐PROEX. E.P. declares no competing interests. S.W.F. declares researcher‐led grants from the National Institute for Health Research, Office of Health Improvement and Disparities, and Novo Nordisk and support for attending academic conferences and events from Novo Nordisk, Eli Lilly, UK Parliament, Welsh Parliament, and Safefood. D.S.C. declares researcher‐led grants from the Spanish Ministry of Science and Innovation. S.S.A. declares funding for equipment and support for attending academic conferences and events from the CNPq. T.M.B.C. declares researcher‐led grants for support for attending academic conferences and events from the CNPq. And M.F.L. declares researcher‐led grants for publication and for support for attending academic conferences and events from the CNPq and FAEPA.

## Supporting information


**Table S1:** Exploratory (EFA) and confirmatory (CFA) factor analysis for the 14‐item FPS‐SF: Standardised factor loadings (initial solution).
**Table S2:** Mean and standard deviation (SD) of the FPS‐SF scores by demographics and factors related to WS.
**Table S3:** Linear regression of factors related to weight change, treatment and concerns related to WS.
**Table S4:** Linear regression results for factors associated with FPS from the 14‐item of FPS‐SF.

## Data Availability

The data that support the findings of this study are openly available in Open Science Framework at https://osf.io/z5t76/, reference number DOI 10.17605/OSF.IO/Z5T76.
