# Supplementary material for: Misconceptions About Obesity and Weight Stigma in Brazilian Healthcare Professionals
Source: Clin Obes. 2026 Mar 18;16(2):e70074. doi: 10.1111/cob.70074 (PMC12999358; doi:10.1111/cob.70074)
Supplement: Supplementary file 1 — Table S1: Exploratory (EFA) and confirmatory (CFA) factor analysis for the 14‐item FPS‐SF: Standardised factor loadings (initial solution). Table S2: Mean and standard deviation (SD) of the FPS‐SF scores by demographics and factors related to WS. Table S3: Linear regression of factors related to weight change, treatment and concerns related to WS. Table S4: Linear regression results for factors associated with FPS from the 14‐item of FPS‐SF. [file COB-16-e70074-s001.pdf]

Supplementary Information

Misconceptions About Obesity and Weight Stigma in Brazilian Healthcare Professionals

Paula Victoria Sozza[a,b,c], Eva Penelo[d], Stuart William Flint[e], David Sánchez-Carracedo[b], Sebastião Sousa Almeida[a], Telma Maria Braga Costa[a,c], Maria Fernanda Laus[a,f]

[a] Departamento de Psicologia, Universidade de São Paulo, Ribeirão Preto, Brasil.

[b] Departament de Psicologia Clínica i de la Salut, Universitat Autònoma de Barcelona, Bellaterra, Barcelona, Spain.

[c] Curso de Nutrição, Universidade de Ribeirão Preto, Ribeirão Preto, Brasil.

[d] Departament de Psicobiologia i de Metodologia de les Ciències de la Salut. Universitat Autònoma de Barcelona, Bellaterra, Barcelona, Spain.

[e] School of Psychology, University of Leeds, Leeds, West Yorkshire, United Kingdom.

[f] Departamento de Ciências da Saúde, Universidade de São Paulo, Ribeirão Preto, Brasil.

Departamento de Psicologia, Universidade de São Paulo, Ribeirão Preto, Brasil

Av. Bandeirantes, 3900, Campus USP (Universidade de São Paulo), Vila Monte Alegre, Ribeirão Preto, SP, Brasil. 14040-900. Telephone number: +55 1633154391.

Email: paula.victoria.silva@usp.br

Table S1. Exploratory (EFA) and confirmatory (CFA) factor analysis for the 14-item FPS-SF: standardized factor loadings (initial solution)

	CFA (1-factor)	EFA (1-factor)	EFA (2-factor)	
Indicator			F1	F2
FAT_PHOBIA1	**.559**	**.558**	**.552**	−.042
FAT_PHOBIA2	**.602**	**.628**	**.622**	−.033
FAT_PHOBIA3	**.672**	**.651**	**.648**	−.168
FAT_PHOBIA4	**.652**	**.634**	**.632**	−.167
FAT_PHOBIA5	**.689**	**.655**	**.653**	−.181
FAT_PHOBIA6	**.703**	**.686**	**.684**	−.182
FAT_PHOBIA7	**.746**	**.715**	**.716**	−.219
FAT_PHOBIA8	**.436**	**.453**	**.450**	.006
FAT_PHOBIA9	**.468**	**.487**	**.491**	.210
FAT_PHOBIA10	.244	.254	.253	.008
FAT_PHOBIA11	**.699**	**.685**	**.681**	−.103
FAT_PHOBIA12	**.430**	**.441**	**.437**	−.055
FAT_PHOBIA13	**.462**	**.481**	**.514**	**.501**
FAT_PHOBIA14	**.512**	**.534**	**.596**	**.650**

In bold: factor loadings > .30

Table S2. Mean and standard deviation (SD) of the FPS-SF scores by demographics and factors related to WS

Variable		*Mean (SD)*
Gender	Women	3.25 (0.68)
	Men	3.57 (0.84)
Provide care for PLWO	Yes	3.28 (0.72)
	No	3.38 (0.65)
Profession	Physician/Doctor	3.62 (0.74)
	Dietitian	3.02 (0.65)
	Psychologist	3.33 (0.72)
	Nursing professional	3.3 (0.69)
	Complementary HCPs	3.56 (0.70)
	Other	3.28 (0.69)
Weight status	Underweight or Normal weight (BMI < 25 kg/m^2)	3.28 (0.75)
	Overweight (BMI between 25 and < 30 kg/m^2)	3.29 (0.72)
	Obesity (BMI > 30 kg/m^2)	3.37 (0.69)
Age group	< 25 years	3.16 (0.72)
	25-34 years	3.32 (0.75)
	35-44 years	3.29 (0.66)
	45-54 years	3.41 (0.77)
	> 55 years	3.39 (0.70)
Q2: Cause of overeating	Emotional/comfort eating	3.15 (0.64)
	Food environment	3.59 (0.73)
	Food addiction	3.18 (0.73)
	Malfunction physiological mechanisms	3.24 (0.79)
	Other	3.33 (0.69)
Q3: Individual commitment prevents obesity	Yes	3.38 (0.73)
	No	3.09 (0.69)
Q4: Individual commitment cures obesity:	Yes	3.40 (0.72)
	No	3.02 (0.73)
Q6: Difficulty to lose weight	Individual lack of motivation/self-discipline sedentary lifestyle	3.46 (0.76)
	Not modifiable [genetic and/or metabolism]	3.04 (0.75)
	Overabundance of products promoting weight gain	3.30 (0.62)
	Other	3.16 (0.73)
Q7: Treatment most effective for severe obesity	Lifestyle interventions [diet, exercise]	3.34 (0.77)
	Medications	3.46 (0.85)
	Psychological support and behavioral modifications	3.17 (0.67)
	Surgery	3.55 (0.74)
Q12: Cause for not losing significant weight while participating in a lifestyle intervention programme	Poor compliance due to lack of motivation/self-discipline	3.69 (0.77)
	Poor compliance beyond patient's control	3.22 (0.71)
	Inadequate aspects by care providers	3.23 (0.70)
	Biological mechanisms of obesity	3.28 (0.73)
Q13: Cause for regaining weight after losing from a lifestyle programme	Individual's diet and lifestyle choices	3.73 (0.74)
	Factors beyond patient's control	3.14 (0.65)
	Inadequate aspects by care providers	3.26 (0.75)
	Relapse/progression of the disease	3.34 (0.69)

Table S3. Linear regression of factors related to weight change, treatment, and concerns related to WS

Outcome	Effects	*B* (95% CI)	*p*[a]
Stigma[b]	Weight changes (reference category: Lost weight)		*.373*
	No changes	−0.02 (−0.18; 0.15)	.832
	Gained weight	0.09 (−0.08; 0.26)	.309
Stigma[c]	Obesity treatment: No	−0.11 (−0.28; 0.06)	.211
Stigma[d]	Weight concerns (reference category: Not concerned about weight and have no plans to lose weight in the next 6 months[e])		*.328*
	Concerned about weight but have no plans to lose weight in the next 6 months	0.08 (−0.20; 0.35)	.597
	Aware of being overweight and seriously considering taking steps to lose weight	0.08 (−0.13; 0.30)	.451
	Aware of being overweight and intending to take steps to lose weight in the next month	0.25 (0.00; 0.50)	.048
	Involved in or enrolled in a weight loss plan	0.21 (0.00; 0.42)	.046
	Have managed to lose weight in the last year and have kept it off	0.21 (−0.03; 0.45)	.090
	Have managed to lose weight in the last year but have not been able to keep it off	0.13 (−0.17; 0.43)	.405

Note. CI: confidence interval.

[a] In italics: test for the overall effect of categorical predictors with more than two categories.

[b] adjusted for all demographics (gender, care provision for PLWO, profession, weight status, and age group).

[c] adjusted for care provision for PLWO and weight-status.

[d] adjusted for gender, profession, and weight status.

[e] Unlike the other regression analyses, in this case the category considered less fatphobic was selected as the reference category.

Table S4. Linear regression results for factors associated with FPS from the 14-item of FPS-SF

Outcome	Effects	*B* (95% CI)	*p*[a]
Stigma	Gender: Women	−0.22 (−0.38; −0.06)	.007
	Provide care for PLWO: Yes	−0.16 (−0.29; −0.04)	.009
	Profession (reference category: Physician/Doctor)		*<.001*
	Dietitian	−0.49 (−0.69; −0.28)	< .001
	Psychologist	−0.30 (−0.53; −0.07)	.011
	Nursing professional	−0.21 (−0.45; 0.03)	.081
	Complementary HCPs	−0.03 (−0.25; 0.19)	.814
	Other	−0.33 (−0.53; −0.13)	.002
	Weight status (reference category: BMI < 25 kg/m^2)		*.410*
	Overweight (BMI between 25 and < 30 kg/m^2)	−0.08 (−0.23; 0.07)	.283
	Obesity (BMI > 30 kg/m^2)	0.02 (−.013; .017)	.829
	Age group (reference category: aged < 25 years)		*.789*
	25-34 years	0.09 (−0.10; 0.27)	.369
	35-44 years	0.03 (−0.16; 0.23)	.735
	45-54 years	0.13 (−0.10; 0.36)	.278
	> 55 years	0.08 (−0.16; 0.33)	.491
Stigma[b]	Q2: Cause of overeating (reference category: Emotional/comfort eating)		*.026*
	Food environment	−0.21 (−0.38; −0.03)	.020
	Food addiction	−0.01 (−0.34; 0.32)	.937
	Malfunction physiological mechanisms	−0.22 (−0.37; −0.07)	.004
	Other	−0.04 (−0.45; 0.36)	.828
Stigma[c]	Q3: Individual commitment prevents obesity: No	−0.23 (−0.37; −0.09)	.001
Stigma[c]	Q4: Individual commitment cures obesity: No	−0.27 (−0.41; −0.13)	< .001
Stigma[c]	Q6: Difficulty to lose weight (reference category: Individual lack of motivation/self-discipline sedentary lifestyle)		*.001*
	Not modifiable [genetic and/or metabolism]	−0.34 (−0.50; −0.18)	< .001
	Overabundance of products promoting weight gain	−0.03 (−0.19; 0.13)	.697
	Other	−0.17 (−0.36; 0.02)	.079
Stigma[d]	Q7: Treatment most effective for severe obesity (reference category: Lifestyle interventions [diet, exercise])		*.031*
	Medications	0.07 (−0.23; 0.34)	.661
	Psychological support and behavioral modifications	−0.08 (−0.23; 0.07)	.286
	Surgery	0.17 (−0.02; 0.35)	.073
	[e] Q7 (reference category: Surgery)		*.031*
	Psychological support and behavioral modifications	−0.25 (−0.41; −0.08)	.003
Stigma[f]	Q12: Cause for not losing significant weight while participating in a lifestyle intervention programme (reference category: Poor compliance due to lack of motivation/self-discipline)		*.007*
	Poor compliance beyond patient's control	−0.34 (−0.54; −0.15)	.001
	Inadequate aspects by care providers	−0.28 (−0.51; −0.05)	.018
	Biological mechanisms of obesity	−0.32 (−0.55; −0.09)	.007
Stigma[c]	Q13: Cause for regaining weight after losing from a lifestyle programme (reference category: Individual's diet and lifestyle choices)		*< .001*
	Factors beyond patient's control	−0.46 (−0.65; −0.27)	< .001
	Inadequate aspects by care providers	−0.36 (−0.55; −0.17)	< .001

Relapse/progression of the disease	−0.32 (−0.52; −0.11)	.003

Note. CI: confidence interval; PLWO: People living with obesity; HCPs = Healthcare professionals; BMI = Body Mass Index; The detailed means and SDs are provided in Table S3.

[a] In italics: test for the overall effect of categorical predictors with more than two categories

[b] adjusted for all demographics (gender, assists, profession, weight status, and age group); category "gluttony" excluded because of extreme low endorsement (*n* = 5)

[c] adjusted for profession

[d] adjusted for gender, care provision for PLWO, and profession

[e] given that the overall effect was statistically significant, an additional pair-wise comparison was conducted, with Surgery as the reference category

[f] adjusted for gender, care provision for PLWO, profession, and weight status
